# Molecular interaction of a putative inhibitor with bacterial SHV, an enzyme associated with antibiotic resistance

**DOI:** 10.1098/rsos.221458

**Published:** 2023-02-08

**Authors:** Shazi Shakil, Syed M. Danish Rizvi, Nigel H. Greig

**Affiliations:** ^1^ King Fahd Medical Research Center, King Abdulaziz University, Jeddah, Saudi Arabia; ^2^ Department of Medical Laboratory Technology, Faculty of Applied Medical Sciences, King Abdulaziz University, Jeddah, Saudi Arabia; ^3^ Center of Excellence in Genomic Medicine Research, King Abdulaziz University, Jeddah, Saudi Arabia; ^4^ Department of Pharmaceutics, College of Pharmacy, University of Hail, Hail, Saudi Arabia; ^5^ Translational Gerontology Branch, Intramural Research Program, National Institute on Aging, National Institutes of Health, Baltimore, MD 21224, USA

**Keywords:** antibiotic resistance, bacteria, computational screening, molecular dynamics, SHV

## Abstract

Tackling the ever-looming threat of antibiotic resistance remains a challenge for clinicians and microbiologists across the globe. Sulfhydryl variable (SHV) is a known bacterial enzyme associated with antibiotic resistance. The SHV enzyme has many variants. The present article describes identification and molecular interaction of a putative inhibitor with the bacterial SHV enzyme as a step towards novel antibacterial drug discovery. The MCULE-platform was used for screening a collection of 5 000 000 ligand molecules to evaluate their binding potential to the bacterial SHV-1 enzyme. Estimation of pharmacokinetic features was realized with the aid of the ‘SWISS ADME’ tool. Toxicity-checks were also performed. The docked complex of ‘the top screened out ligand’ and ‘the bacterial SHV-1 protein’ was subjected to molecular dynamics simulation of 101 ns. The obtained ligand molecule, 1,1'-(4H,8H-Bis[1,2,5]oxadiazolo[3,4-b:3′,4'-e]pyrazine-4,8-diyl)diethanone, displayed the most favourable binding interactions with bacterial SHV-1. A total of 15 amino acid residues were found to hold the ligand in the binding site of SHV-1. Noticeably, 12 of the 15 residues were found as common to the binding residues of the reference (PDB ID: 4ZAM). The RMSD values plotted against the simulation time showed that nearby 11 ns, equilibrium was reached and, thenceforth, the ‘SHV-1-Top ligand’ complex remained typically stable. Starting from around 11 ns and straight to 101 ns, the backbone RMSD fluctuations were found to be confined inside a range of 1.0–1.6 Å. The ligand, 1,1′-(4H,8H-Bis[1,2,5]oxadiazolo[3,4-b:3′,4′-e]pyrazine-4,8-diyl)diethanone, satisfied ADMET criteria. Furthermore, the practicability of the described ‘SHV-1-Top ligand’ complex was reinforced by a comprehensive molecular dynamics simulation of 101 ns. This ligand hence can be considered a promising lead for antibiotic design against SHV-1 producing resistant bacteria, and thus warrants wet laboratory evaluation.

## 1. Introduction

Tackling the ever-looming threat of antibiotic resistance remains a challenge for clinicians and microbiologists worldwide [1–5]. The stock of effective antibiotic armory available to clinicians continues to shrink. In fact, this scenario can be described as a continuing race between the scientists and the bacteria, or as a battleground between two armies [6–9]. Whereas scientists keep designing novel antibiotics, bacteria outsmart them by developing novel resistance strategies. SHV (sulfhydryl variable) constitutes a bacterial enzyme associated with antibiotic resistance, and it has numerous variants. The molecular architecture of SHV-1 comprises two domains. One of the domains is totally α-helical. The other domain consists of a five-stranded antiparallel β-sheet. The beta sheet is in turn surrounded by α-helices on both sides [10]. SHV-1 shares 68% of its amino acids with TEM-1, which is among the best studied bacterial enzymes [11]. Ser70, Lys73, Ser130, Glu166, Asn170 and Lys234 are regarded as the six catalytically important residues of SHV-1 [10]. Another noteworthy feature is the *Ω Loop*. It extends from amino acid residues 160–181. This stretch of amino acid residues has a role in influencing substrate spectra of SHV-1 [10]. Emergence of antibiotic resistance due to the production of SHV enzyme variants in bacteria is an established phenomenon. In the past, the SHV bacterial enzyme has been implicated in some notable outbreaks, like in Mexico [12]. Recent studies have also drawn attention to SHV mediated bacterial infections [13]. A 2021 study reported a high rate of multidrug resistance in *Pseudomonas aeruginosa* isolates, and bacterial SHV was among the significant resistance enzymes [14]. Presently, 228 SHV-variants have been reported [15]. It is understandable that finding a permanent solution to halt the emergence of antibiotic resistance in bacteria has not yet been achievable; however, scientists can increase the pace of drug discovery research to design new antibiotics or identify modifications to existing drug structures to restore effectiveness. Recently, we proposed a molecular lead against CTX-M-15, which is another important bacterial enzyme implicated in the antibiotic resistance phenomenon [2]. Researchers have additionally reported promising hits aimed at blocking OXA variants [16]. Similarly, research for identifying improved inhibitors against the bacterial SHV enzyme is warranted to thwart antibiotic resistance threats mediated via this mechanism. Computational screens buoyed by analyses of simulation trajectories have proved to be a crucial element within current drug-discovery workflows [2,17,18]. In an interesting study, the authors used *in silico* techniques to investigate the drug resistance mechanism of pyrazinamidase (PncA) enzyme in *Mycobacterium tuberculosis* [19]. It was found that the mutation (D8G, S104R and C138Y) in PncA was responsible for rigid binding cavity which in turn abolished conversion of pyrazinamide to its active form and was the reason for pyrazinamide resistance [19]. Similarly, docking and molecular dynamics simulation techniques have been used to study other diseases as well [20–23]. In the current scenario, artificial intelligence based technologies have started to enter the field of antibiotic design. This is expected to accelerate the pace of antibiotic discovery [24,25]. Therefore, the significance of *in silico* studies stands justified.

The rationale of this study is best described by the ‘*Thumb Rule for Antibiotic Design’* against bacteria [26]. The rule states that ‘*the minimum pace of drug design ought to match the swiftness with which bacteria display cutting-edge resistance mechanisms; thereby outwitting the antibiotics and, in turn, the researchers*' [26]. SHV is among the most important enzymes that impart antibiotic resistance to bacteria. Hence, the objective of this work was to identify a promising lead for antibiotic design against SHV-1 producing bacteria.

## 2. Material and methods

### 2.1. Examining the binding site

The entry with the accession code 4ZAM (resolution: 1.42 Å; R-Value Free: 0.193; R-Value Work: 0.166; R-Value Observed: 0.168) in the protein data bank was selected as the reference complex for the present work [27]. It represents the *reality* crystal of a relatively new antibiotic, AVIBACTAM bound to the active pocket of SHV-1 bacterial enzyme. The three-dimensional structure of the binding spot in the above-mentioned complex was studied with the aid of Discovery Studio Visualizer [BIOVIA] as well as YASARA Structure v. 21.8.27.

### 2.2. Molecular screening

The drug discovery platform ‘MCULE’ was employed to perform molecular screening of an adequately large ligand collection [28]. The ‘SMILES’ of the reference molecule (as present in the active site of the reference crystal 4ZAM) was submitted to ‘Chemspider Structure Search’ that subsequently produced a ligand-properties table with the aid of ‘Percepta Platform’. Grounded on this properties-table, the ‘maxima and minima input values' required to be supplied to the MCULE workflow builder were determined and used in the screening protocol [28]. A total of 5 000 000 ligand molecules were examined to appraise their binding potential to bacterial SHV-1 enzyme. Briefly, a single violation of Lipinski's ‘rule of five’ was tolerated, thereby providing some span to the initial filters. The input against the maximum number of allowed rotatable bonds was selected as 7. A mass range of 220–300 Da was entered. The range for polar surface area of the putative inhibitors was provided as 115–170 Å^2^. Likewise, the assigned ranges for ‘H-bond donors’ and that for ‘H-bond acceptors’ were entered as 0–5 and 5–10, respectively. The ‘sampler size’ was kept as 1000, whereas ‘the maximum number of compounds after sphere exclusion’ was assigned a value of 3 000 000. A similarity threshold of 0.85 was used in the molecular screening workflow. The molecular descriptors were determined with the aid of ‘Open Babel Linear Fingerprint’. The other default values were left untouched.

### 2.3. Molecular docking of bacterial SHV-1 with test ligands

The three-dimensional protein structure (bacterial SHV-1 enzyme in pdb format) was retrieved from PDB ID 4ZAM [27] using the Discovery Studio Visualizer [BIOVIA]; and the same was supplied to the workflow builder of the MCULE-platform. The afore-mentioned platform executed docking by VINA [29]. It is noteworthy that the MCULE platform used the latest AutoDock Vina v. 1.2.0 [30]. A molecular docking grid of 60 Å^3^ was used. A semi-flexibile docking approach was used whereby the test ligands were kept flexible and the target protein was treated as a rigid molecule. The *x*, *y* and *z* values (as required to specify the position of the docking grid) were entered into the workflow as −15.7633, −7.0655 and −3.5709, respectively. These coordinates were, in turn, determined by careful examination of the reference structure [PDB ID: 4ZAM; 27] by the visualizer. The default grid spacing value of 1.0 was used.

### 2.4. VINA docking scores and pharmacokinetic features

The test ligands were graded by ‘VINA docking scores’ [29]. Accordingly, the ligands that constituted the upper layer by VINA were noted. Delineation of the pharmacokinetic features of the selected ligands was realized with the aid of the ‘SWISS ADME’ tool [31]. A range of evaluations, namely, ability or inability to cross the Lipinski, Ghose, Veber, Egan, Muegge, PAINS plus Brenk filter, were performed for each test ligand. The afore-mentioned filters principally evaluate the promise of the candidate ligands to act as future drugs [32–36]. Hence, the ligands that demonstrated upper VINA docking scores and, in addition, successfully crossed a minimum 5 of these filters were noted.

### 2.5. VINA docking score cut off, zero RO5 criterion, BBB non-permeability and safety profile for humans

A set of 40 ligands that constituted the top stratum of the VINA output was subjected to advance exploration. Candidate inhibitors that demonstrated a ‘VINA docking score’ >−7.0 kcal mol^−1^ were disqualified. The choice of this value (−7.0 kcal mol^−1^) was totally arbitrary. It was done merely to exclude the lower ranking ligands and narrow down the number for further filtration steps. The residual collection of the molecules was reduced further, as follows. Only those molecules were retained that displayed absolutely zero violation of the well-known ‘RO5’ rule. Next, any of the molecules determined to be ‘Blood-Brain Barrier-permeable’ was rejected. Toxicity assessments were duly performed for the remainder of the ligands with the aid of the ‘toxicity checker’ functionality within the online drug discovery platform [28].

### 2.6. Zero violation of ‘lead-likeness’ and ‘synthetic accessibility score cut off’

The non-toxic ligand set obtained above was assessed by the criterion of ‘Zero violation of Lead-Likeness'. Further, the ligands that displayed a synthetic accessibility score > 2.55 by SWISS ADME were rejected [31]. Ligand structures were also evaluated for their total number of rotatable bonds. As a final point, a candidate SHV-inhibitor that exhibited overall maximum suitable features as per the presented drug-discovery workflow (and, strikingly, demonstrated ‘Zero RO5 violation’, ‘Zero violation of Lead-Likeness' along with ‘No toxicity for humans’) was selected as the ‘Top anti-SHV ligand’ in this research work.

### 2.7. ‘Ligand interactions’ and ‘molecular overlay’ analyses for the ‘SHV-1-top ligand’ complex

The amino acid residues crucial to hold the ‘Top anti-SHV ligand’ within the active site of the bacterial enzyme were labelled by Discovery Studio Visualizer. Furthermore, the ‘SHV-1-top ligand’ complex was overlaid upon the PDB structure used as the reference in this study [PDB ID: 4ZAM; 27]. The ‘Molecular Overlay’ was achieved by the Discovery Studio Visualizer.

### 2.8. Molecular dynamics simulation of 101 ns by YASARA STRUCTURE

The licensed v. 21.8.27, (C)1993–2019 of YASARA STRUCTURE was used to run 101 ns molecular dynamics simulation for the docked complex of ‘the top screened out ligand’ and ‘the bacterial SHV-1 protein’ across three replicas [37]. The simulation protocol was initialized with the optimization of hydrogen bonds. The p*K*a value corresponding to the pH value of 7.4 was calculated [38]. Next, 0.9% NaCl ions were added to the system. The simulation cell was neutralized followed by energy minimization. This aided geometry-correction of the input structure. In order to remove conformational stress, a ‘short steepest descent minimization’ procedure was implemented. Furthermore, the afore-mentioned procedure was sustained with the aid of simulated annealing. The simulated annealing, in turn, used a time step of 2 fs. The atom velocities were scaled down by 0.9 at every tenth step until the system attained convergence. Having performed the above-mentioned early experimental processes, the core molecular dynamics simulation was executed for 101 ns. It was noteworth that the simulation protocol was performed with the aid of AMBER14 [39] meant for the solute, and with GAFF2 [40] along with AM1BCC [41] for the ligand. The dynamics protocol used the TIP3P water model. This dynamics system employed a default temperature of 298 Kelvin and a pressure of one atmosphere. Explicit details of the algorithms can be obtained from Krieger and Vriend 2015 [42]. Analysis of simulation trajectory was achieved by using a ‘macro’ named ‘md_analyze.mcr’. This macro was packed with the specific commands meant for trajectory analysis in the YASARA STRUCTURE. The program used a specific language, named YANACONDA, for performing complex tasks. Publication quality images (importantly, the graph for RMSD versus simulation time) were generated. Snapshots were retrieved after each 250 ps interval. Consequently, a total of 405 snapshots were obtained and then carefully examined.

## 3. Results and discussion

The importance of computational studies lies in the fact that these studies reduce the burden of total number of experiments needed to be performed in wet laboratory [43]. The pace of antibiotic design is expected to take a significant leap with the emergence and advancement of artificial intelligence based technologies [24,25]. Antibiotic resistant bacteria pose a serious threat to the treatment of various types of infections including the very serious ones, like diabetic foot ulcers. Hence, the importance of design/discovery of novel compounds to tackle the continued threat of antibiotic resistant bacteria is understandable. CTX-M, SHV, TEM and OXA are some of the most important enzymes that impart antibiotic resistance to bacteria. Previously, we have reported a lead compound against bacterial CTX-M-15 [2]. Likewise, in the present study we have used SHV-1 as the target.

### 3.1. The binding site

Examination of the three-dimensional structure of the binding spot in the 4ZAM complex crystal by Discovery Studio Visualizer [BIOVIA] and further by YASARA Structure version 21.8.27 revealed that the protein-ligand interactions of the binding site involved a total of 15 residues. Specifically, these amino acid residues were Met 69, Ser 70, Lys 234, Ser 130, Gly 236, Ala 237, Thr 235, Arg 244, Thr 167, Asn 132, Glu 166, Asn 170, Lys 73, Val 216 and Tyr 105.

### 3.2. Outcome of molecular screening

**Figure 1.**
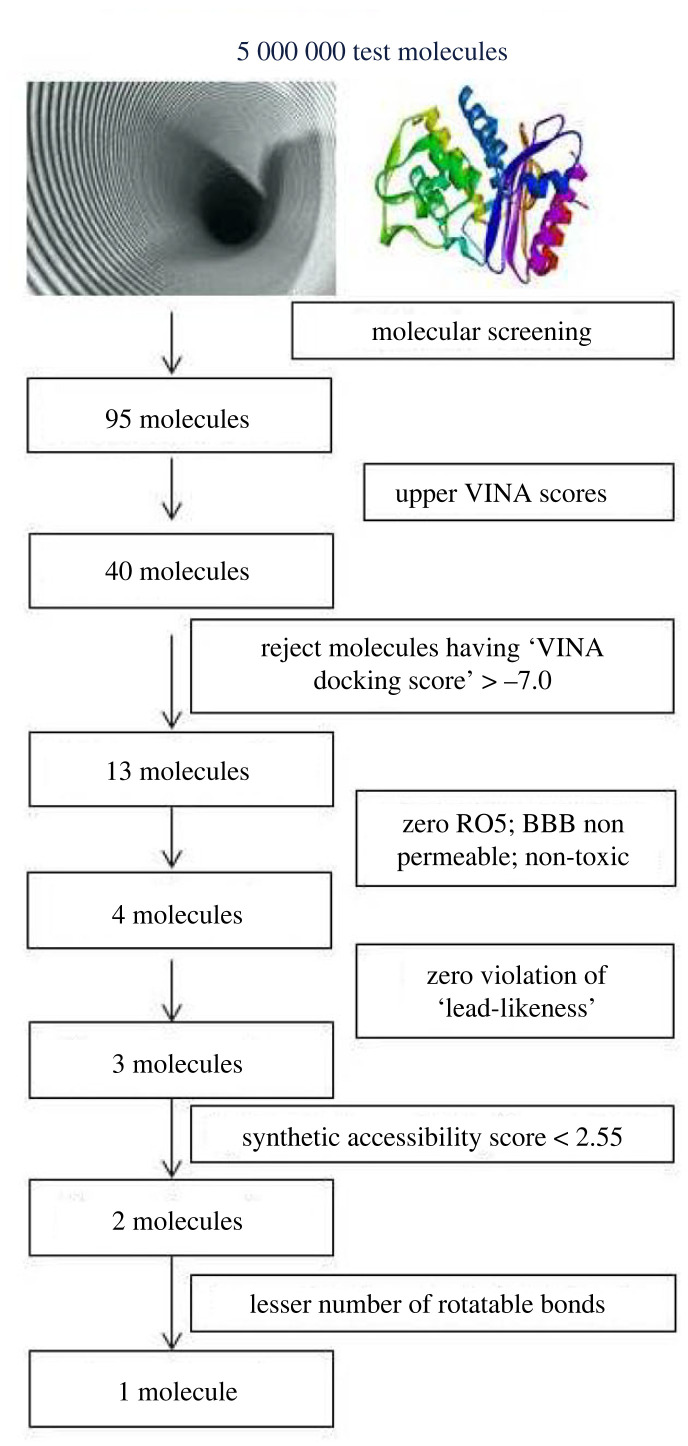
A flowchart representing the step-by-step protocol followed to screen 5 000 000 putative inhibitors of bacterial SHV-1 enzyme.

The screening protocol was based on the three-dimensional structure of the bacterial SHV-1 enzyme. In the present study, a total of 5 000 000 ligand molecules were examined to appraise their binding potential to bacterial SHV-1 enzyme, which resulted in a collection of 95 candidate inhibitors (figure 1).

### 3.3. Ranking of ligands by ‘VINA’ and results of ‘SWISS ADME-profiling’

An array of successfully published articles focused on protein-ligand interactions ignited our interest in the search and design of inhibitors targeting bacterial proteins that impart antibiotic resistance [2,17,18,44]. Based on this, we have extensively worked in the field of antibiotic resistance in bacteria [3,5–9]. In this study, ‘VINA’ was relied upon to accomplish SHV-1-inhibitor screening (implemented within the MCULE platform). The above-mentioned program remains particularly noteworthy as it is acknowledged for its power to improve the general precision of a molecular docking experiment [29]. Accordingly, VINA stands tall among the many available programs used for computational screening of large collections of molecules. The top ‘VINA-docking-scores’ generally indicate a finer ‘fit’ of select small molecules within the targeted active site. In this manner, a set of 40 test molecules that constituted the top stratum of the VINA output was acquired and subjected to additional examination.

### 3.4. Additional filtration

#### 3.4.1. VINA docking score cut off, zero RO5 criterion, BBB non-permeability and safety profile for humans

The candidate SHV inhibitors that exhibited a docking score of more than −7.0 kcal mol^−1^ by VINA were disqualified. Accordingly, 13 molecules were left for additional filtration. These molecules were analysed by SWISS ADME to interrogate their pharmacokinetic characteristics [31], as ligands predicted to express favourable pharmacokinetic features are obviously carried forward to successive levels of drug discovery cascades. It is noteworthy that in the early stage of the molecular screening workflow, a molecule that succeeded in passing five of the seven filters, namely Lipinski, Ghose, Veber, Egan, Muegge, PAINS and Brenk filters was highlighted for additional examination [32–36]. This imparted due flexibility to the screening cascade at the initial level. Likewise, a single violation of Lipinski's ‘rule of five’ was tolerated, thereby providing some wideness to the starting filters; albeit, the afore-mentioned set of 13 putative SHV-1 inhibitors was reduced further by rationally selected stringent criteria. As detailed in the Methodology section, stringency was increased as only those ligands (from among the 13 inhibitors) that displayed absolutely zero violation of the well-known ‘RO5’ rule were retained. Thereafter, any of these 13 molecules predicted to be ‘Blood-Brain Barrier-permeable’ was rejected. Further, toxicity assessments were also performed utilizing the ‘toxicity checker’ functionality within the online drug discovery platform [28]. As a result, only four molecules remained. Table 1 presents the pharmacokinetic features of these four upper scoring presumed inhibitors of bacterial SHV-1 enzyme.

**Table 1.** Pharmacokinetic features of the four upper scoring presumed inhibitors of bacterial SHV-1.

features	MCULE-3577694120-0-51	MCULE-6950463386-0-12	MCULE-2993388113-0-16	MCULE-3665465035-0-11
IUPAC name	1,1'-(4H,8H-bis[1,2,5]oxadiazolo[3,4-b:3′,4'-e]pyrazine-4,8-diyl)diethanone	1-(4-fluorophenyl)-5-[(1H-tetrazol-5-ylmethyl)sulfanyl]-1H-tetrazole	5,7-dihydroxy-2-(methylamino)thieno[3,2-b]pyridine-3-carbonitrile	4-[(1H-benzotriazol-1-ylmethyl)amino]-1,2,5-oxadiazole-3-carboxylic acid
formula	C_8_H_6_N_6_O_4_	C_9_H_7_FN_8_S	C_9_H_7_N_3_O_2_S	C_10_H_8_N_6_O_3_
molecular mass	250.17	278.27	221.24	260.21
consensus log P_O/W_	0.23	1.26	1.12	0.50
RO5 violations	0	0	0	0
hydrogen bond acceptors	8	7	3	7
hydrogen bond donors	0	1	3	2
rotatable bonds	2	4	1	4
TPSA (Å²)	121.84	123.36	117.15	118.96
molar refractivity	54.94	63.06	58.49	62.43
gastro intestinal absorption	high	high	high	high
BBB permeable	no	no	no	no
Log S (Ali))	−1.75	−3.51	−3.05	−3.50
Lipinski-filter	yes; 0 violation	yes; 0 violation	yes; 0 violation	yes; 0 violation
Ghose-filter	yes	yes	yes	yes
Veber-filter	yes	yes	yes	yes
Egan-filter	yes	yes	yes	yes
Muegge-filter	yes	yes	yes	yes
PAINS-filter	0 alert	0 alert	0 alert	0 alert
Brenk	0 alert	0 alert	0 alert	0 alert
synthetic accessibility	2.53	2.49	2.65	2.97
lead-likeness	yes	yes	no; 1 violation: MW < 250	yes

**Figure 2.**
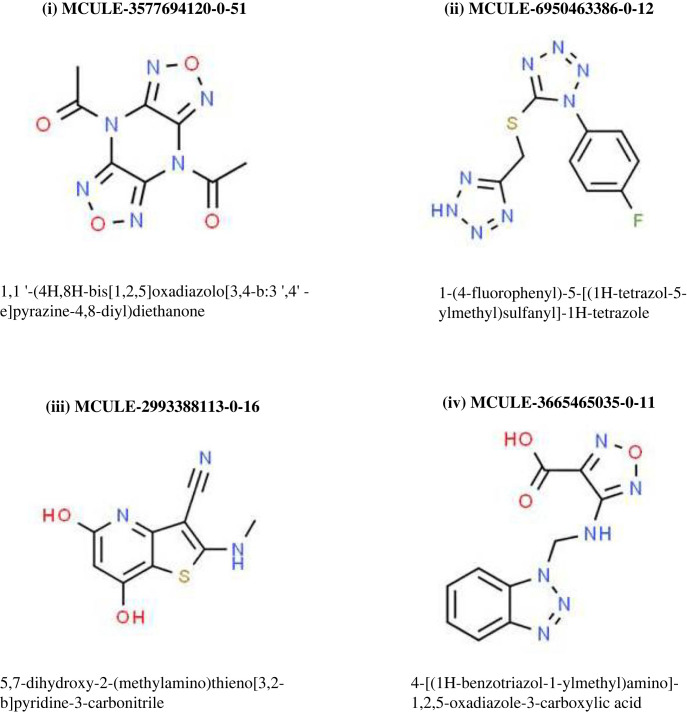
Chemical structures of the four upper scoring presumed inhibitors of bacterial SHV-1.

These four postulated inhibitors of bacterial SHV-1 were found to possess identifiers as MCULE-3577694120-0-51, MCULE-6950463386-0-12, MCULE-2993388113-0-16 and MCULE-3665465035-0-11. Figure 2 shows the chemical structures of the four upper scoring presumed inhibitors of bacterial SHV-1.

#### 3.4.2. Zero violation of ‘lead-likeness’ and ‘synthetic accessibility score cut off’

Notably, each of the above-mentioned four presumed inhibitors proved successful in crossing seven of seven filters [32–36, table 1]. However, one molecule (MCULE-2993388113-0-16) was found to display one violation of ‘lead-likeness’ (MW < 250) leading to its rejection. Accordingly, three molecules remained. Further increasing the stringency of the screening cascade, any ligand displaying a synthetic accessibility score greater than 2.55 was rejected, thereby sparing only two molecules (MCULE-3577694120-0-51 and MCULE-6950463386-0-12) to be considered further as putative SHV-1-inhibitors. In the computational drug design cascades, candidate molecules that possess lower numbers of rotatable bonds are generally preferred. The two ligands, MCULE-3577694120-0-51 and MCULE-6950463386-0-12, were found to have two and four rotatable bonds, respectively. Hence, MCULE-3577694120-0-51 was preferred over the latter, and was subjected to advanced investigations described in the subsequent sections. The ‘SMILES’ notation of the ligand MCULE-3577694120-0-51 was entered into CHEMSPIDER to generate its IUPAC name. In this manner, 1,1'-(4H,8H-Bis[1,2,5]oxadiazolo[3,4-b:3′,4'-e]pyrazine-4,8-diyl)diethanone was the top molecule that emerged from the screening cascade (figure 2, molecule (i)). This molecule displayed a high predicted gastrointestinal absorption (as per SWISS ADME program), a plus point for probable ability to be administered orally. Furthermore, MCULE-3577694120-0-51 was considered to be non-BBB permeable; another positive feature for a future antibiotic in order to minimize potential off-target binding and neurotoxicity. Therefore, MCULE-3577694120-0-51 or 1,1'-(4H,8H-Bis[1,2,5]oxadiazolo[3,4-b:3′,4'-e]pyrazine-4,8-diyl)diethanone was designated as the ‘Top anti-SHV-1 ligand’ in the present study.

#### 3.4.3. Outcome of ‘ligand interactions’ and ‘molecular overlay’ analyses for the ‘SHV-1-Top ligand’ complex

**Figure 3.**
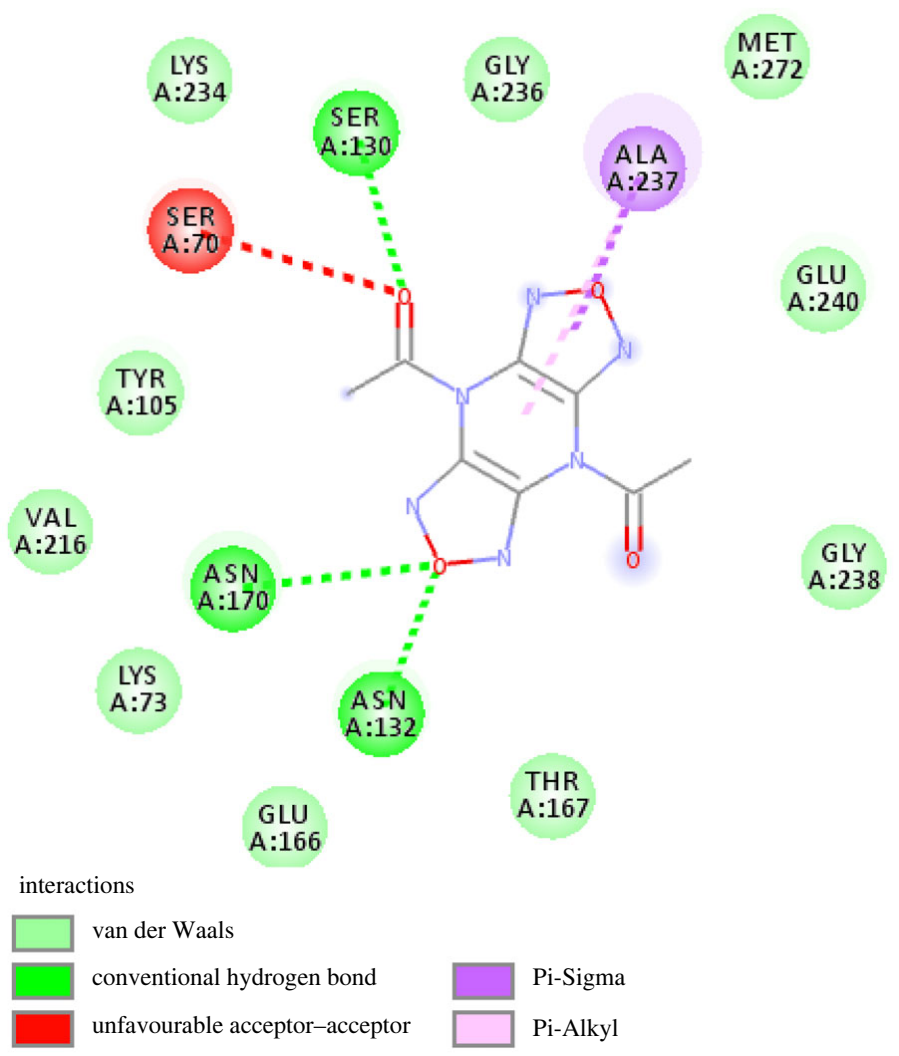
‘Two-dimensional-Diagram’ of the ‘SHV-1-Top ligand’ complex generated with the aid of ‘Discovery Studio Visualizer’ [BIOVIA].

Figure 3 presents the ‘Two-dimensional-Diagram’ for the ‘SHV-1-Top ligand’ complex created with the aid of ‘Discovery Studio Visualizer’ [BIOVIA].

**Figure 4.**
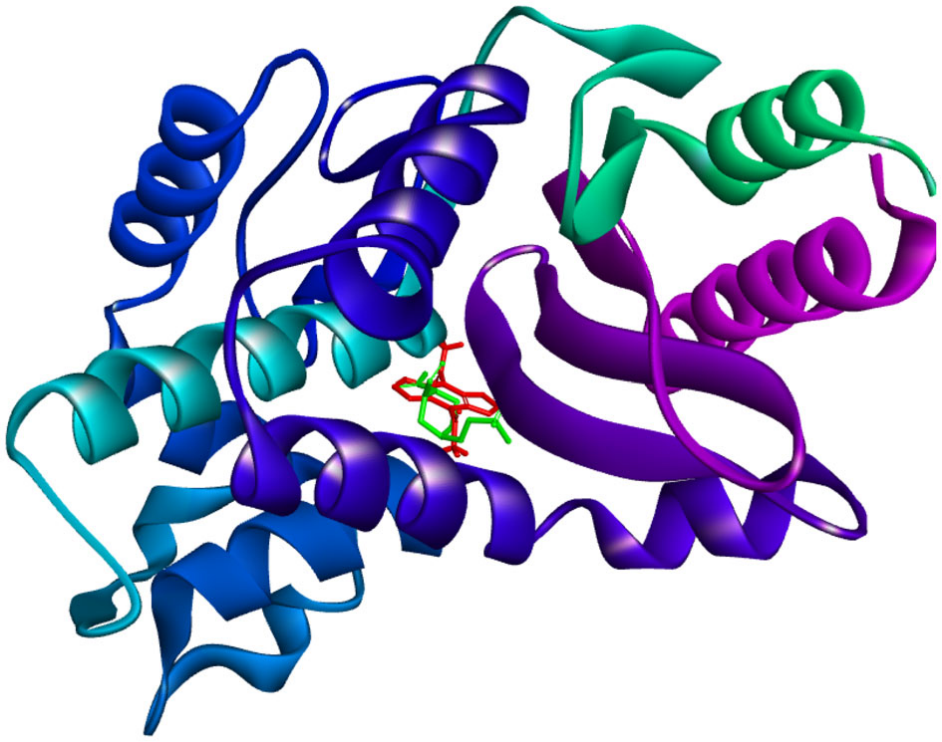
‘Molecular Overlay’ depiction of the ‘SHV-1-Top ligand’ complex paralleled against the ‘4ZAM reference crystal’. The ‘Top anti-SHV-1 ligand’ and the ‘reference ligand’ are shown as ‘stick models’ in red and green colours, respectively.

The amino acid residues crucial to hold the ‘Top anti-SHV-1 ligand’ within the active site of the bacterial enzyme are labelled. The ‘Top anti-SHV-1 ligand’, 1,1'-(4H,8H-Bis[1,2,5]oxadiazolo[3,4-b:3′,4'-e]pyrazine-4,8-diyl)diethanone was found to interact with the bacterial antibiotic resistance enzyme via 15 amino acid residues. Notably, 12 of the 15 amino acid residues were found to match with the binding contact residues within the 4ZAM reference crystal. The common residues were affirmed as Ser 70, Lys 234, Ser 130, Gly 236, Ala 237, Thr 167, Asn 132, Glu 166, Asn 170, Lys 73, Val 216 and Tyr 105. Significantly, the binding spot for avibactam, i.e. the small molecule present in the reference crystal [PDB ID: 4ZAM; 27], was confirmed to be the same as that of the ‘SHV-1-Top ligand’ complex; as also indicated by the afore-mentioned 12 shared residues. Moreover, the ‘SHV-1-Top ligand’ complex was paralleled against the 4ZAM crystal (used as reference) by the ‘molecular overlay’ tool of Discovery Studio Visualizer (BIOVIA). Figure 4 shows the ‘Molecular Overlay’ depiction for the ‘SHV-1-Top ligand’ complex paralleled against the ‘4ZAM reference crystal’ [27].

It is evident from the above figure that the ‘top ligand’ and the ‘reference ligand’ bind at the same site (figure 4). This further confirms the accuracy of the docking experiment.

#### 3.4.4. Outcome of molecular dynamics simulation of 101 ns by YASARA STRUCTURE

**Figure 5.**
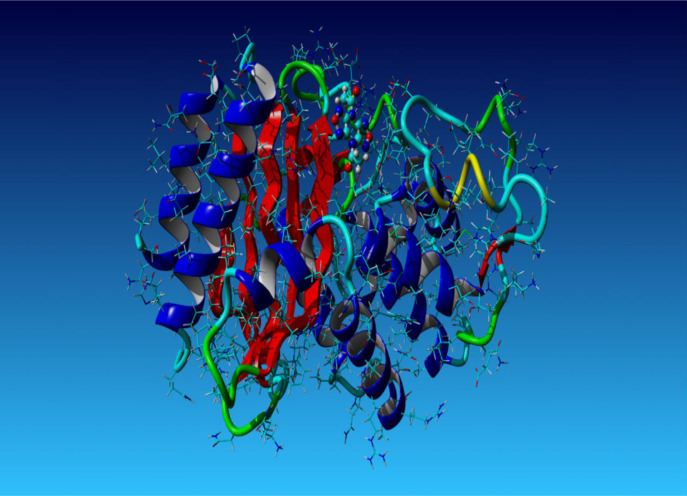
The ‘ray-traced diagram’ of the ‘SHV-1–Top ligand’ complex that entered the simulation system.

**Figure 6.**
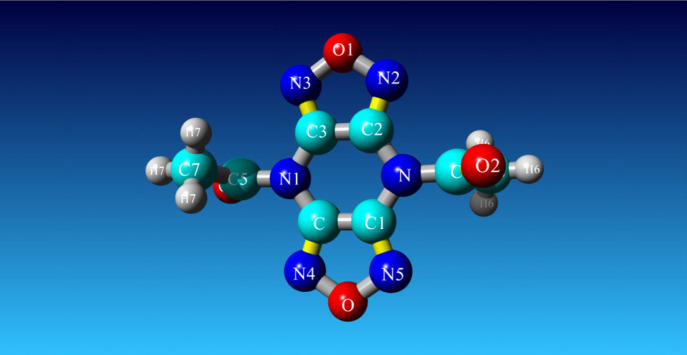
The ‘ray-traced diagram’ showing the ‘Top anti-SHV-1 ligand’.

**Figure 7.**
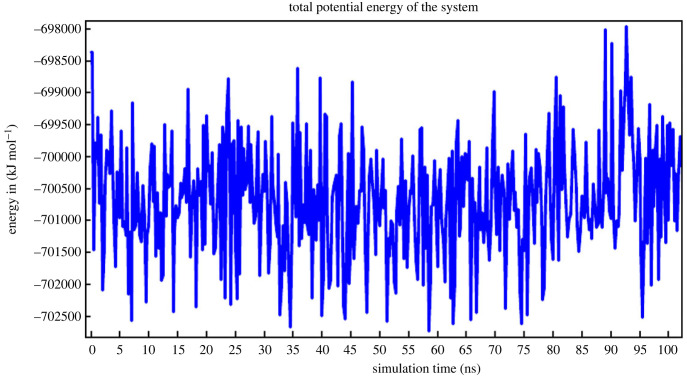
The fluctuations of the total potential energy of the system as the simulation progressed.

The ‘SHV-1-Top ligand’ complex harbouring the screen-selected inhibitor 1,1'-(4H,8H-Bis[1,2,5]oxadiazolo[3,4-b:3′,4'-e]pyrazine-4,8-diyl)diethanone within the binding pocket of SHV-1 bacterial enzyme was subjected to 101 ns molecular dynamics simulation. This took approximately 71 h for completion. Analysis of simulation trajectory was undertaken with the aid of AMBER14 implemented within YASARA STRUCTURE v. 21.8.27. As the snapshots were retrieved after each 250 ps interval, the macro command ‘md_analyze.mcr’ extracted 405 snapshots from the simulation run. The process of simulation was repeated three times. This fully affirmed the results. Many diagrams and plots of interest, namely the ‘ray-traced diagram’ of the ‘SHV-1-Top ligand’ complex that entered the simulation system (figure 5); one more ‘ray-traced diagram’ showing the ‘Top anti-SHV-1 ligand’ (figure 6) and additionally the figure displaying the fluctuations of the total potential energy versus simulation time (figure 7), were also constructed.

Specifically, the simulated system shown in the above figure was composed of one protein molecule having 265 residues (4073 protein atoms), one ligand molecule having 24 atoms, 44 Na ions, 44 Cl ions and 15 698 water molecules (figure 5).

Bonds present in the chemical structure of the ‘Top anti-SHV-1 ligand’ are shown in specific colours according to the bond orders (gray = 1, blue = 1.25, red = 1.5 and lime green = 2.5) (figure 6).

**Figure 8.**
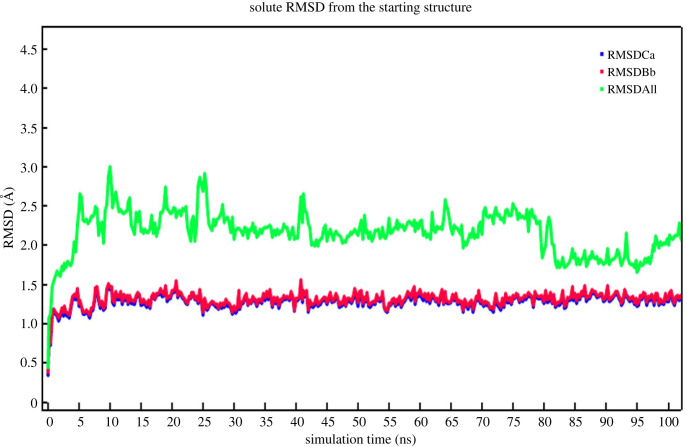
‘Solute RMSD from the starting structure’ plotted against the ‘simulation time’.

Generally, when a molecular dynamics simulation gets started from an energy-minimized ‘frozen’ conformation, a significant increase in energy is observed during the first picoseconds. This is because the added kinetic energy is partly stored as potential energy. Moreover, on a larger time-frame, the potential energy may not decrease, a common reason being the counter ions. These are originally located at the positions having the lowest potential energy, typically close to charged solute groups. Subsequently, they detach to gain entropy and potential energy. The above plot shows that as the simulation progressed, the total potential energy of the system fluctuated approximately in a range of *−*702 500 kJ mol^−1^ to −698 000 kJ mol^−1^ (figure 7). Most importantly, a graph for ‘Solute RMSD from the starting structure’ versus ‘simulation time’ was generated as shown below (figure 8).

The RMSD values plotted against the simulation time demonstrate that nearby 11 ns, equilibrium was reached and, thenceforth, the ‘SHV-1-Top ligand’ complex remained typically stable. Starting from approximately 11 ns and continuing to 101 ns, the backbone RMSD fluctuations were found to be confined within a range of 1.0–1.6 Å. This narrow range appeared acceptable for the RMSD plot (figure 8).

Additional plots like the one displaying the radius of gyration of the solute along the progress of simulation and the one depicting ligand conformation RMSD versus simulation time were also generated. These plots are available as electronic supplementary material (electronic supplementary material, figure S1 and S2. Significantly, the ligand conformation RMSD values were found to fluctuate within a slender range of 0.5 to 1.6 Å, thereby further confirming the stability of the ligand in the binding pocket of the bacterial enzyme. Overall, the screening cascade data coupled with the above-described simulation plots are indicative of the efficacy of the screen-selected ligand against SHV-1 bacterial enzyme, as well as the feasibility of the proposed ‘SHV-1-Top ligand’ complex.

Limitations of the study: it is important to mention that the results presented in this article are purely based on computational experiments. Further validation by wet laboratory experiments is required.

## 4. Conclusion

The ligand, 1,1'-(4H,8H-Bis[1,2,5]oxadiazolo[3,4-b:3′,4'-e]pyrazine-4,8-diyl)diethanone, which was selected from 5 000 000 test molecules by a computational screening cascade, satisfied ADMET criteria. Furthermore, the practicability of the described ‘SHV-1-Top ligand’ complex was reinforced by a comprehensive YASARA molecular dynamics simulation of 101 ns. In this light, the suggested ligand emerges as a promising lead for antibiotic design against SHV-1 producing resistant bacteria. The ligand is worthy of wet laboratory investigative study.

## Data Availability

The data are provided in the Dryad Digital Repository [45]. Supplementary material is available online [46].
